# Flying Ad Hoc Networks: A New Domain for Network Communications

**DOI:** 10.3390/s18103571

**Published:** 2018-10-21

**Authors:** Antonio Guillen-Perez, Maria-Dolores Cano

**Affiliations:** Department of Information and Communication Technologies, Universidad Politécnica de Cartagena, 30202 Cartagena, Spain; mdolores.cano@upct.es

**Keywords:** drone, unmanned aerial vehicle (UAV), flying ad hoc network (FANET), mobility models, positioning algorithms, propagation models, radiation pattern, WiFi

## Abstract

The advent of flying ad hoc networks (FANETs) has opened an opportunity to create new added-value services. Even though it is clear that these networks share common features with its predecessors, e.g., with mobile ad hoc networks and with vehicular ad hoc networks, there are several unique characteristics that make FANETs different. These distinctive features impose a series of guidelines to be considered for its successful deployment. Particularly, the use of FANETs for telecommunication services presents demanding challenges in terms of quality of service, energy efficiency, scalability, and adaptability. The proper use of models in research activities will undoubtedly assist to solve those challenges. Therefore, in this paper, we review mobility, positioning, and propagation models proposed for FANETs in the related scientific literature. A common limitation that affects these three topics is the lack of studies evaluating the influence that the unmanned aerial vehicles (UAV) may have in the on-board/embedded communication devices, usually just assuming isotropic or omnidirectional radiation patterns. For this reason, we also investigate in this work the radiation pattern of an 802.11 n/ac (WiFi) device embedded in a UAV working on both the 2.4 and 5 GHz bands. Our findings show that the impact of the UAV is not negligible, representing up to a 10 dB drop for some angles of the communication links.

## 1. Introduction

Unmanned aerial vehicles (UAVs), also known as drones, have been adopted in many sectors such as agriculture, wildfire monitoring, border surveillance, or telecommunications, to name a few [1,2]. Simple taxonomies can be used to categorize UAVs, for instance, in terms of the type of flight (autonomous or remotely controlled), their size (large or small), the type of wings, or their communication capabilities. Regarding the types of wings, there are two main categories: fixed-wing UAVs (FW-UAVs) and rotary-wing UAVs (RW-UAVs). Whereas FW-UAVs present longer flight times, higher flight speeds, and a more aerodynamic design, RW-UAVs are able to perform vertical take-off and landing (VTOL), exhibit greater stability (can control yaw, pitch, roll, and throttle), and have the capacity to hover over static points. A brief comparison between FW-UAVs and RW-UAVs is presented in Table 1. Regarding the communication capabilities, we differentiate between single UAVs, which usually employ star topologies to communicate with a base station (BS) or a satellite [3], and multi-UAV systems [4]. Nowadays, most civil and military applications are implemented using multi-UAV systems that consist of a swarm or formation of small UAVs. This approach brings several advantages, such as lower times to complete tasks, cost reduction, higher scalability, and more reliability, among others [5,6,7], and is known under the term flying ad hoc network (FANET).

FANETs can be seen as a subset of the well-known concept of mobile ad hoc networks (MANETs). MANETs are networks composed of mobile devices such as laptops, cellular phones, sensors, etc. Similarly to vehicle ad hoc networks (VANETs), whose components are cars, buses, ambulances, etc., incorporating embedded communication devices, in FANETs, UAVs are wirelessly interconnected, either directly or using intermediate nodes. Thus, only a small set of nodes need to be connected to the BS/satellite. Even though it is clear that FANETs share common features with MANETs and VANETs, there are several unique characteristics that makes them different, namely, mobility, topology changes, radio propagation, and energy constraints.

The mobility of the nodes in a FANET is usually greater than in VANETs and MANETs. The speed of FANETs nodes is highly variable, from being static in aerial coverage or in relaying network-nodes to flying at full speed in search and rescue tasks. More importantly, these nodes have the peculiarity of being able to move in the three spatial dimensions (3-D) with total freedom of movement and RW-UAVs can rotate independently on the three axes (roll, pitch, and yaw). This high mobility of the nodes in FANETs is very different from that presented by the nodes in MANETs and VANETs. In MANETs, nodes usually have very low mobility (e.g., people walking at 6 km/h [8]) and limited speed changes. In VANETs, nodes correspond to vehicles circulating in the streets, presenting medium speed changes (around 100 km/h on highways and around 50 km/h on urban roads [9]) in a 2-D mobility plane (horizontal plane). Nevertheless, speed in VANETs could be higher than in FANETs in some particular cases [10]. These differences have a direct impact on the proposed mobility models. Since these models are employed to simulate the network in more realistic scenarios, it is expected to resemble the way the nodes move. For instance, the random walk mobility model is used in MANETs when the direction and the speed of the nodes are chosen randomly. As another example, the street random walk model or the Manhattan mobility model are used in VANETs because nodes are only allowed to move through streets or highways. Most of these models for MANETs and VANETs are close to reality since the movements of the nodes are very predictable and are also limited by the layout of roads. However, mobility models adopted in MANETs and VANETs cannot be applied directly in FANETs. It is necessary to propose more adequate models that represent the behavior of UAVs considering their high 3-D mobility. As a consequence of the higher mobility degree of the nodes in FANETs, network topology changes are also more frequent in FANETs than in MANETs and VANETs. Other factors that also influence topology changes in FANETs are the speed of the nodes, changes in distances among nodes, fast changes in the quality of the communication links, and the (likely) reduced life time of the nodes due to energy limitations and/or failures. In traditional MANETs and VANETs, these circumstances are not always present.

**Figure 1:**
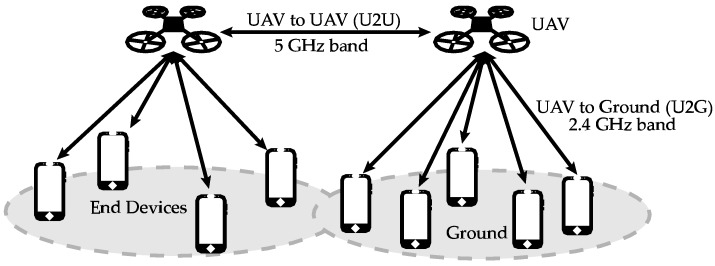
An example of communication links in a FANET using the 5 GHz band for U2U communication and the 2.4 GHz band for U2G coverage.

On the other hand, the model that predicts propagation losses for electromagnetic waves in FANETs is different from the model used in MANETs and VANETs. In MANETs and VANETs, loss models usually assume that there is not line of sight (nLoS) between the transmitter and the receiver and that there are a large number of reflections from environmental obstacles and diffraction of buildings. On the contrary, FANET nodes are flying at a considerable height, away from possible reflections that may originate in the ground, in buildings, or obstacles. Moreover, there is usually line of sight (LoS) between the interconnected FANET nodes. Due to the position of the nodes in FANETs, two types of links with different characteristics can be distinguished (see Figure 1): a link that connects two flying nodes (UAV-to-UAV or U2U) and a link that connects a flying UAV and a terrestrial node (UAV-to-Ground, UAV-to-BS, or U2G). LoS is predominant in U2U links. In U2G links, if there is LoS then the direct and reflected components in the ground will predominate. Otherwise, if there is nLoS then there will be a large number of components due to reflections and diffraction. Therefore, the model of propagation losses should take into account for these two types of links, resulting in a different approach compared to propagation models used in MANETs and VANETs. Table 2 summarizes the main differences among MANETs, VANETs, and FANETs.

Particularly for the telecommunications field, UAVs could be used to provide WiFi coverage through an aerial network (e.g., in emergency situations) or to substitute wireless base stations when these are not operational, among other examples. There are several works in the related literature that tackle communication challenges in FANET. For instance, some studies address the communication characteristics of UAVs and others evaluate the received signal level in order to obtain an approximate channel model that could predict propagation losses [11,12,13,14,15,16]. However, most of these studies do not usually take into account the particular radiation pattern (RP) of the communication device embedded in the UAV, do not include a study of the RP in a controlled environment (e.g., an electromagnetic simulator software, such as 4NEC2 (Numerical Electromagnetic Code) or CST (Computer Simulation Technology), or an anechoic chamber), do not take into account the orientation of the communication module antennas, or even simplify the RP to an isotropic one. The study of the RP in a controlled environment would allow a more effective deployment of UAVs in a real environment. The reason lies on the fact that with that knowledge, we could optimize the power radiated by the antennas and thus better results in terms of throughput, signal level, and battery lifetime could be achieved.

In this work, we present a comprehensive survey on FANETs, focusing on three related topics: mobility models, positioning algorithms, and propagation models. Numerous proposals have been introduced by researchers to address these challenging themes. We describe, classify, and qualitatively compare those proposals, suggesting a clear taxonomy for each topic. Then, we contribute to the advance of the state of the art by measuring the impact of an unmanned aerial vehicle on the on-board WiFi radiation pattern. To do so, we conduct a set of experiments in a controlled environment, a radio-frequency (RF) anechoic chamber, to obtain the RP of a WiFi communication module embedded in a UAV. From the results, we quantify the effect (if any) of the UAV engines (revolutions per minute (RPM) and vibrations produced in the chassis and antennas), the UAV propellers, and the UAV chassis on the RP. In light of these outcomes, we show the conditions that should be met to assume that an UAV has an isotropic RP, or (at least) a constant RP. Therefore, we provide a basis to simplify radiation studies and to obtain more accurate experimental results in future related works.

The rest of the paper is organized as follows. In Section 2 the revision of the state of the art is presented and discussed. The case study is detailed in Section 3. The paper ends with a conclusion in Section 4.

## 2. State of the Art

The review is organized in three subsections addressing mobility models, positioning algorithms, and propagation models. All these topics have a clear impact on the completion of FANET as mentioned in the introduction section.

### 2.1. Mobility Models

A mobility model represents the movement of the nodes of a network, i.e., the changes in their position, speed, and acceleration over time in a delimited scenario. Mobility allows the nodes to adapt to the needs of each application, offering great dynamism and better performance depending on the selected mobility model. In addition, researchers can simulate FANETs with more realism using mobility models. Since nodes in FANETs are highly mobile, the choice of an appropriate mobility model for each simulation scenario is essential to evaluate the performance of the network before a real deployment to obtain results as real as possible [20]. We classify the existing mobility models into five different classes: pure randomized, time-dependent, path planned, group, and hybrid.

**Figure 2:**
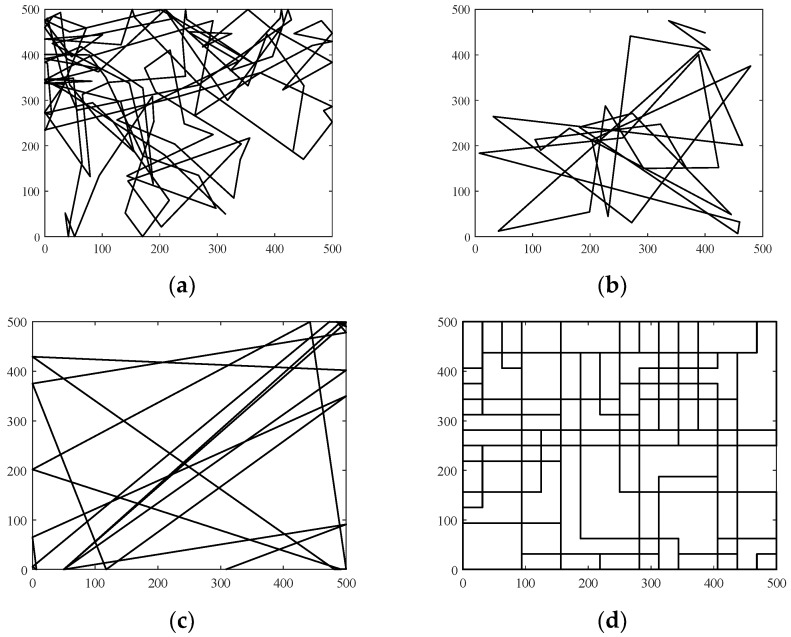
Pure randomized mobility trajectories: (**a**) Random Walk mobility model where each node selects a random direction [0, 2π) and speed *S* [*Smin*, *Smax*] that is maintained for a predefined time interval *t* or a constant distance *d* (500 × 500 m, *t* = 5 s); (**b**) Random Waypoint (*Tpause* = 3 s); (**c**) Random Direction, as in RWP but the node searches the edge point of the simulation area; and (**d**) Manhattan Grid with example of an urban scenario with 16 horizontal and 16 vertical streets.

**Figure 3:**
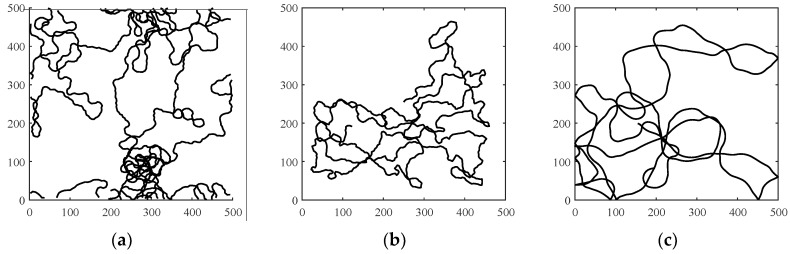
Time-Dependent mobility trajectories: (**a**) Boundless Simulation Area, (**b**) Gauss-Markov, and (**c**) Smooth Turn.

**Figure 4:**
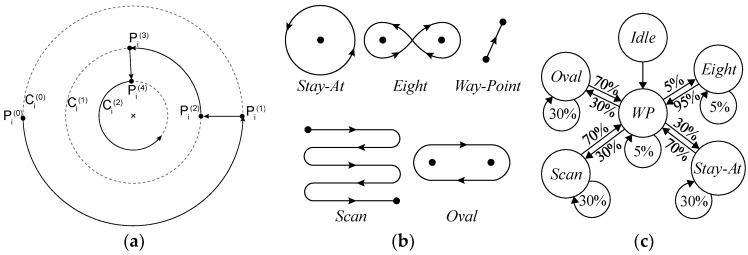
Path-Planned mobility trajectories: (**a**) Semi-Random circular mobility model; (**b**) Paparazzi autopilot UAV Movements (*Stay-At*, *Eight*, *Way-Point*, *Scan*, and *Oval*); and (**c**) PPRZM state machine.

**Figure 5:**
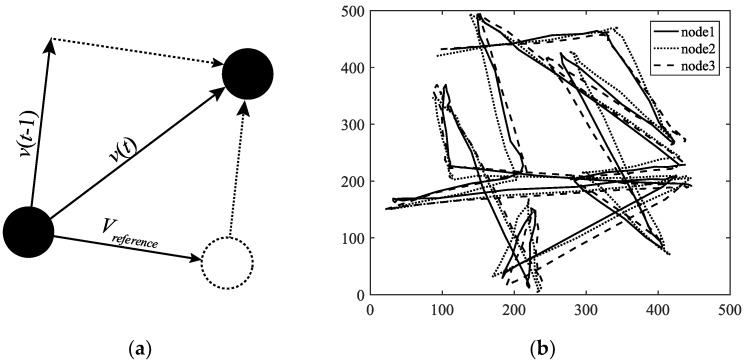
Column mobility models: (**a**) Example of a single step in PSMM; (**b**) Trajectory simulation using RPGM.

**Figure 6:**
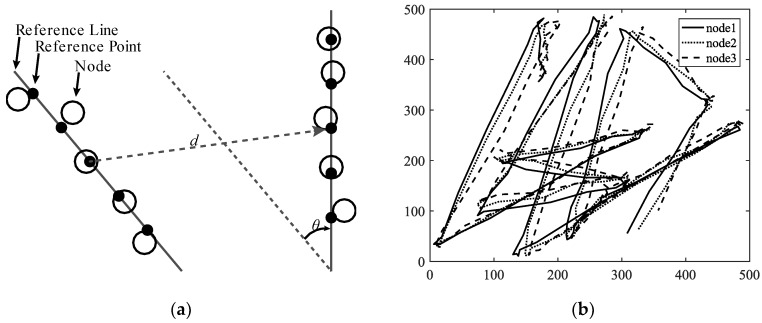
Column mobility models: (**a**) Example of a single step in CLMN where each node has its reference point and reference points are placed on a reference line that moves a distance *d* and rotates an angle *θ*; (**b**) Trajectory simulation of three nodes using CLMN where reference points randomly move through the simulation area and each node moves randomly around its reference point.

**Figure 7:**
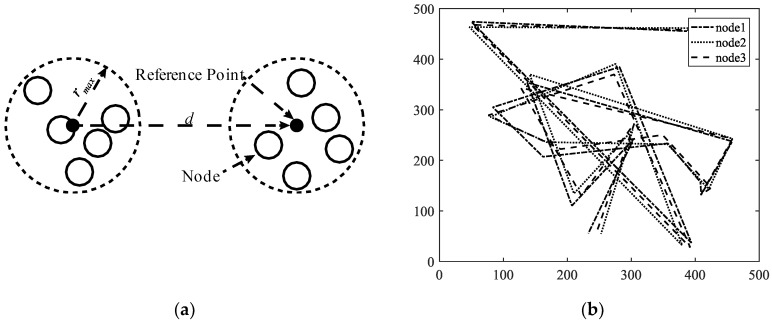
Column mobility models: (**a**) Example of a single step in Nomadic Community (NC) with five nodes where the nodes have a maximum distance *r_max_* to move away from the reference point and the reference point moves through the simulation area a random distance *d* following a randomized model; (**b**) Trajectory simulation of three nodes using NC.

**Figure 8:**
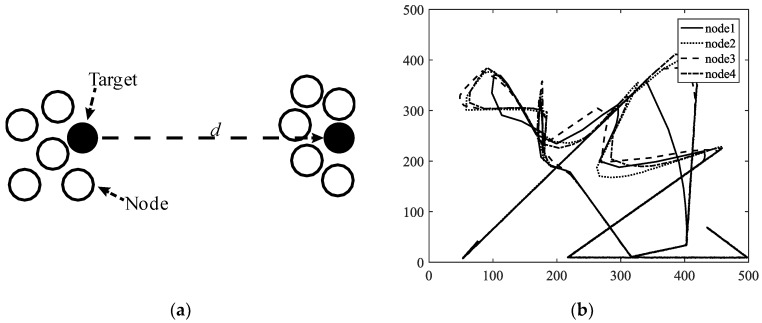
Column mobility models: (**a**) Example of a single step in PRS with a target node (black) moving a distance *d* and it is pursued by five purser nodes (white); (**b**) Trajectory simulation of a target node and three pursuer nodes using PRS.

- Pure Randomized Mobility Models: Random mobility models are the simplest and most common models to simulate the movement of nodes in an ad hoc network. Each node randomly selects its direction, speed, and time, independently of other nodes. In this group, the following mobility models stand out: Random Walk [21], Random Waypoint [17], Random Direction [22], and Manhattan Grid [23]. Figure 2 graphically depicts the characteristics of these well-known models. For instance, in the Random Waypoint mobility model (RWP), each node randomly chooses both its destination within the simulation area and the speed *S* between [*Smin*, *Smax*]. After this, the node starts moving at that constant speed towards the destination, and once the node reaches its destination, it remains static for a time *Tpause*, as shown in Figure 2b. Given that the RWP mobility model tends to group the nodes in the center of the simulation area, a node using the random direction (RD) mobility model searches for the edge of the area, remains static when arrives, and then selects a new direction angle (Figure 2c).
- Time-Dependent Mobility Models: Movement is based on mathematical equations that depend on both the instant when the simulation is calculated and the state of each node to avoid sudden changes in speed and direction. The most prominent examples in this category are: Boundless Simulation Area [24], Gauss-Markov [25], Enhanced Gauss-Markov [26], 3-D-Gauss-Markov [27], and Smooth Turn [19]. The main disadvantage of the Boundless Simulation Area (BSA) mobility model (see Figure 3a) is that when a node disappears along an edge, it reappears on its opposite edge, which could be undesirable. On the other hand, the Gauss-Markov (GM) mobility model (see Figure 3b) is more realistic than the RW, RWP, and RD models, but it does not allow for modeling the behavior of FW-UAV, especially turns. The Enhanced Gauss-Markov mobility model (EGM) is a modification of the GM model specifically proposed for FANETs. The results obtained with this model are similar to the behavior that a FW-UAV would have while flying, since there are no sudden stops or sharp turns within the simulation. In addition, the EGM has a progressive boundary-avoidance mechanism to avoid edges, which allows soft curves at the boundaries. The three-dimensional Gauss-Markov mobility model (3D-GM) is another modification of the GM model designed for FANETs with the main characteristic of including mobility in the three dimensions, eliminating the bounce generated at the edges of the simulation area, as in the EGM model. Last, in the Smooth Turn (ST) mobility model (see Figure 3c), a node randomly selects a point along the line perpendicular to its heading direction and flies in circles around that point for a random time.
- Path-Planned Mobility Models: These mobility models are characterized by nodes following a pre-calculated trajectory without taking any random direction. The nodes are forced to follow a series of movement patterns, only changing randomly between the different patterns that each model has. Within this category we have the Semi-Random Circular Movement [28] and the Paparazzi [18] models. The former (SRCM) is a suitable model to simulate the behavior of UAVs that are flying over a point in circles with different radius collecting information, as shown in Figure 4a. The latter (PPRZM) is a stochastic mobility model based on the Paparazzi autopilot software for UAV [29]. This Paparazzi autopilot presents five possible movements: *Stay-At*, *Eight*, *Way-Point*, *Scan*, and *Oval* (represented in Figure 4b). Thus, the PPRZM mobility model is based on a state machine where each state is one of the possible movements that a UAV can do. The state machine is showed in Figure 4c.
- Group Mobility Models: They are based on the fact that the different nodes of the network move together around a common point. This behavior has great advantages, especially in the field of FANETs, where this approach can be used in many applications. Three representative examples can be found in this category, namely, Exponential Correlated Random [30], Particle Swarm [31], and Reference Point Group Mobility [32]. The Exponential Correlated Random (ECR) mobility model is similar to the time-dependent mobility models. To control the groups of nodes a set of variables is added, whose configuration is not straightforward. The Particle Swarm Mobility Model (PSMM) is based on the iterative optimization method Particle Swarm Optimization (PSO) [33]. This model calculates the speed and direction of each node according to the previous speed/direction and the position in which nodes are located with respect to the reference point. The operating principle of PSMM is illustrated in Figure 5a. In the Reference Point Group Mobility (RPGM) model, nodes are grouped into clusters. Each cluster has a center, which can be a logical center or a leading node. The center follows an RWP mobility model and the nodes will be moving around the center (see Figure 5b). This mobility model has numerous variants, the most important ones are Column (CLMN) [21] (see Figure 6), Nomadic Community (NC) [21] (see Figure 7), and Purse [21]. For instance, nodes in the Purse (PRS) mobility model try to catch a target, which may be another node or an imaginary point as represented in Figure 8. As stated by its authors, [21] “you have an apple-pie in your hand and some flies are trying to land on it. You move the pie to avoid them to reach it… Flies are the nodes.”
- Hybrid Mobility Models: This set of hybrid models is usually based on a combination of previous models. As an example, the Hybrid Markov Mobility Model with Pheromones (H3MP) [34] is based on the Markov and the Distributed Pheromone Repel [35] models joining the advantages of both. Whereas Markov chains improve the overall behavior of the UAV, the pheromone approach allows a more precise control of mobility in a local and dynamic way by sharing knowledge among UAVs.

A similar taxonomy was proposed in Reference [36], but including the topology-control category and not including the hybrid one. We believe that the topology-control category is closer to positioning techniques, therefore we opted to limit our taxonomy to the mentioned classes. Some of the mobility models that we reviewed in this section do not take into account characteristics of the RW-UAV and FW-UAV nodes such as variations in speed during turns, rotation angles, smooth trajectories, mechanisms to avoid collisions, etc. Interestingly, some authors have demonstrated that these characteristics may not have a significant impact on the results [37]. That is, it might not be significant differences between realistic models (which consider a smooth trajectory, implement mechanisms to avoid collisions, and include accelerations and realistic speeds), and unrealistic models. Nevertheless, further research is needed to corroborate this fact. Table 3 presents a summary of all mobility models described in this section, including the most appropriate type of UAV for each model and their use.

### 2.2. Positioning Protocols

Coverage in a given area is a classic problem in wireless telecommunication networks. Widely studied, most recent proposals use evolutionary algorithms [38], integer linear programming [39], or greedy algorithms [40] (among others) to plan the best location of base stations and cellular systems. However, these solutions do not lead to an optimal deployment in FANETs due to their unique characteristics. As a consequence, original methods have been presented for optimal positioning of UAV nodes in FANETs, whose objective is either to offer coverage at ground level or to increase the capacity of traditional mobile networks [15,41] or MANETs [42]. The proposed taxonomy is hence divided into two categories: height-based positioning and network-based positioning.

- Height-based positioning. First, there are several studies that analyze the impact of the height of the UAV node on the achieved coverage. In Reference [43], the optimal position of a UAV node that maximizes coverage through a mathematical model is obtained. The channel model on which they based their study contains statistical parameters for the urban environment defined by the International Telecommunication Union (ITU). Thus, their results included a formula to obtain the optimal altitude based on the maximum allowable losses, and some additional statistical parameters. Mozaffari et al. [44] extended the work done in Reference [43]. They obtained the optimum height where to place a UAV to offer the maximum coverage area with the minimum transmitted power. In addition, they studied the use of two UAVs to provide coverage in two scenarios, namely, interference-free and full interference between both UAVs. In the first scenario, the height and distance between UAVs was calculated for optimal deployment in an area. In the second scenario, the existence of interference did not allow full coverage of the entire area, but the results showed the existence of an optimal separation between both UAVs, which provided the highest proportion of coverage area. Nevertheless, the authors used a theoretical channel model, without relying on any of the existing channel models for the U2G communication channel. Similarly, the authors in Reference [45] also investigated the optimal height of a UAV to provide coverage. In this case, they considered that the channel presented path loss and scattering. Particularly, they employed a Rician fading model with dependence on the elevation angle. They showed that there is an optimal position that maximizes the coverage area and presented the lowest losses.
- Network-based positioning. Regarding the control of nodes positioning, there are a large number of studies. As an example, the deployment of a network with FW-UAV is evaluated in [46]. These nodes cannot remain static in a point, but they must be flying over a point. The proposed algorithm dynamically adjusts the position of the nodes and the radius of the UAVs according to the demand of the users at ground level, increasing the performance of the network in terms of the probability of coverage and reduction of the delay. Inspired by the typical movements of bacteria (which move according to their attraction to attractors or away from repellents, aka chemotaxis), the authors in Reference [47] obtained the optimal position of a set of nodes of a FANETs by setting the height of each node, since vertical movements require a greater amount of energy than horizontal movements. In Reference [41], the authors analyzed the optimal deployment of a FANET obtaining the probability of coverage for the UAV–ground link. Then, based on the circle packing theory, the number of available nodes, and the gain and bandwidth of the antennas, they obtained the position and the optimal altitude for each node in an optimal deployment. From a different perspective, Lyu et al. [48] investigated the minimum number of UAVs needed to provide coverage in an area, ensuring that each terrestrial node in the ground has coverage with at least one UAV node. To do so, UAVs were placed following an inward spiral path until all the terrestrial nodes were covered. The authors assumed that the UAV–ground channel presented LoS and they fixed the transmitted power of the UAV to obtain the minimum signal-to-noise ratio (SNR) in the receivers. Another solution was introduced with Self-Deployable Point Coverage (SDPC) [49]. SDPC studies the optimal positioning of FANETs nodes in coverage expansion tasks in cases of disasters, looking for the optimal position to cover the largest number of people per UAV, and maintaining a connection between each UAV. Whereas SDPC can be used with RW-UAVs nodes, its application with FW-UAVs is limited because it does not consider smooth trajectories and progressive curves. The authors in Reference [50] proposed an algorithm to find the optimal position of a UAV based on fine-grained LOS information in a highly urban area. Finally, Chetlur Ravi and Dhillon [51] obtained an expression of the coverage probability for the UAV–ground link modeling the base stations as a uniform binomial point process, and in Reference [52], Sharma et al. employed a neural-based cost function to deploy multiple UAVs in an area with full coverage. Their results showed that they were able to achieve higher capacity than the existing cellular network.

Despite the notable results accomplished in positioning, none of these studies contemplated the impact that the UAVs themselves can have on the embedded communication devices in charge of providing coverage, and therefore likely affecting their outcomes.

### 2.3. Propagation Models

The characteristics of the radio channel through which waves are transmitted are crucial for the design and planification of any communications system. These characteristics allow for mathematically modeling the channels and thus obtaining a propagation model that simulates the attenuation of the waves when they propagate. The simplest propagation model is the Friis free space model [53], which only takes into account the frequency of the signal and the distance, with the corresponding limitations. Planning and deployment in FANETs involve knowing in advanced the characteristic of the radio channel. Calculating the power received in an aerial network from an UAV to a BS (or vice versa) or between two UAVs requires an appropriate channel modelling for each case, considering the unique characteristics of these networks in terms of propagation, such as high possibility of a direct ray (LoS), ground reflection effects, motion effects in 3-D of the UAV (roll, pitch, and yaw), changes in environmental conditions, or variations in distances between nodes, among others. Due to these characteristics, the scientific community has developed a series of theoretical, empirical, and semi-empirical models that approximate a model of channel losses through which electromagnetic waves (EM) are attenuated. The most significant existing body of work on UAV channels modeling is focused on the U2G communication channel (e.g., References [16,54,55,56,57,58,59]), although there are a few works for U2U channels [13,14]. Our categorization is hence divided into four classes: theoretical models, empirical models, semi-empirical models, and well-known models. Whereas the first three categories focus on developing new models, the adequacy of well-known models is studied in the last category. A summary of the reviewed channel models is included in Table 4.

- Theoretical models: Regarding the theoretical models, in Reference [16], the authors proposed a very detailed propagation model of the U2G channel for aerial small cells. This model considered a lot of factors that other theoretical models do not include such as the altitude of the UAV, the particular environments (urban, sub-urban, dense-urban, and urban high-rise), rain (light, moderate, and heavy) multipath fading, LoS probability, shadowing, wave propagation, gaseous absorption, Doppler spread, and other additional losses that may exist. However, this model did not take into account the impact that the UAV can have on the radiation pattern of the communication device. We will see this limitation in all similar studies. Holis et al. [54] proposed another model of theoretical propagation losses for the U2G channel in different types of urban areas in the frequency band between 2 and 6 GHz (since these are some of the frequencies used for mobile systems). In addition, this model considered the elevation angle between the UAV and the terrestrial node, as well as the possibility of LoS. Finally, the model was compared with a set of real measurements showing that the applicability of this propagation model was likely.
- Empirical models: On the other hand, there are empirical models obtained from a series of measurements made in different urban/rural scenarios. Indeed, there is a large number of articles related to obtaining a propagation loss model in an empirical way. For example, in References [55,56] the U2G channel model was obtained from a set of measurements in the 800 MHz band. In Reference [57], the authors made a summary of a U2G measurements campaign. They showed the outcomes obtained and the propagation models calculated in a series of experiments conducted in conjunction with NASA. Khawaja et al. [58] studied the ultrawideband (UWB) propagation models for U2G channels. These UWB models allow signals with a greater bandwidth, a lower attenuation by penetration, and a great co-existence with narrow band signals.
- Semi-empirical models: The semi-empirical models are initiated as theoretical models and then varied according to a set of measures to match reality. In Reference [59], the authors started with a series of measurements of the RSS (received signal strength) of the U2G channel in an urban area, and then selected the propagation model that most resembled the collected measurements. It was Rice + Log-Normal (Loo’s model [60]). Finally, they modified the chosen model such that the results with that model corresponded to the obtained measures. Goddemeier et al. [13] assessed another semi-empirical model, but in this case for the U2U channel. To do this, the authors extended the Rice channel model in the U2U link to take into account the multipath effect of the signals produced by an UAV flying at a fixed altitude. In this study, the antennas of the UAV were placed in a vertical position, which makes the reflections with the ground less frequent and have a lower power. In addition, the impact of the UAV on the transmitted power was not known, since only the horizontal plane of the complete RP was studied.

- Well-known models: There are other works in the related literature that focus on verifying the adequacy of already-known propagation models in FANET scenarios. For example, in Reference [61], Jung et al. proposed a method to select the environment or areas (urban, sub-urban, rural, mountainous, near the sea, etc.) where the air network is going to be deployed in order to choose the U2G channel propagation model that more adapts to reality. The results showed that their method worked for a wide variety of environments. Daniel et al. [62] analyzed the applicability of traditional propagation models to FANETs. For this, the authors made a comparison of different propagation models used for conventional mobile systems (e.g., Cost 231-Hata, Walfish-Ikegami, Erceg, Har, WINNER II B1, C1, C2, and D2) with the ray tracing model. They found out that the WINNER II C1 and Walfish-Ikegami models are suitable options when the air node is below 30 m, otherwise the free space model predicted propagation losses quite well.

As it can be seen, and to the best of the authors’ knowledge, most works addressing the study of propagation models in the state of the art have not considered the influence that the UAV can have on the radiation pattern of the integrated communication device. This can lead to erroneous results in mobility, in positioning, and generally in performance, since either loss due to the UAV chassis/vibrations/etc. are not being considered in the theoretical models, or these losses are being mistakenly included in another factor such as noise in uncontrolled environments. As we will demonstrate later, these losses can suppose a power drop of up to 10 dB in some angles for the U2G link. Therefore, a factor that takes into account the position/orientation of the UAVs and the nodes on the ground should be incorporated in the propagation models to include the losses due to the UAV chassis/vibrations/etc. To assist on this matter, the next section presents a study on the influence of a UAV on the radiation pattern of an embedded communication device. As a result, a representation of the expected losses will be obtained for the different links (U2U and U2G) in all the spatial planes.

## 3. Case Study: Understanding the Impact of Embedded Devices on the Radiation Pattern of UAVs

This case study has three goals. First, we will derive from the measurements the best configuration of the antennas for an isolated WiFi communication device. This WiFi node will be latter embedded in the UAV to provide simultaneous communications in both the 2.4 GHz and the 5 GHz bands. Each band is dedicated to a U2U link and to a U2G link, respectively, as shown in Figure 1. Second, we will get the RP of the whole system: the WiFi device embedded in the UAV. From the results, we will quantify the effect (if any) of the UAV engines (revolutions per minute—RPM—and vibrations produced in chassis and antennas), the UAV propellers, and the UAV chassis on the RP. Third, in the light of these outcomes, we will show the conditions that should be met in order to assume that an UAV has an isotropic RP or (at least) a constant RP, and thus we provide a basis to simplify future related works.

### 3.1. Related Work

Only a few works can be found in the related literature addressing the impact of UAVs on the propagation models. In Reference [63], a set of experiments were performed measuring the RSS in the U2G channel under the standard 802.11 a in the 5 GHz band. In addition, the best position of the antennas in terms of offering a higher level of received signal was inferred. The results showed that when the antennas were horizontal, the yaw differences could be handled, but if the antennas were placed perpendicular to the ground, the signal level varied greatly during the accelerations/decelerations of the UAV. Yanmaz et al. [12] carried out a series of experiments measuring the received power in a U2G communication link. Each communication node employed three transmit antennas in a triangular position in order to eliminate the nulls, thus preventing orthogonal polarization. In the experiments, the ground base station was fixed, and a UAV moved in various scenarios at different heights, orientations, and distances. The main drawback of this work is the lack of control over the environment parameters, such as interferences or reflections, that could alter the results and limits reproducibility. In Reference [64], the authors conducted a series of measurements of the U2G channel in a sub-urban mountainous environment at frequencies of 968 MHz and 5060 MHz. Cheng et al. [65] performed a set of measurements of the signal received from the U2G channel with various antenna positions, various base station heights, and different distances between nodes. They concluded that to obtain the highest throughput and thus the highest RSS, the antennas of both devices must be parallel to each other.

In summary, none of these previous works obtained accurate results given the lack of control over the experimental scenario, limiting the reproducibility and the applicability of the obtained outcomes. Consequently, we decided to study the influence of a UAV on the radiation pattern of an embedded communication device in a fully-controlled environment. The complexity involved in modeling all the physical components of the UAV and the WiFi communication device with a high degree of detail (antennas, carbon fiber chassis, motors, metal parts, vibrations, etc.) in an electromagnetic simulator such as 4NEC2, HFSS, CST, etc., is very high. In addition, results obtained in simulation should be later corroborated with real measurements. Consequently, we skipped this previous step and perform the measurements directly in a RF anechoic chamber under conditions of real use. That is, we carry out the experiments in a fully controlled environment so reproducibility is guaranteed.

### 3.2. Experimental Setup

**Figure 9:**
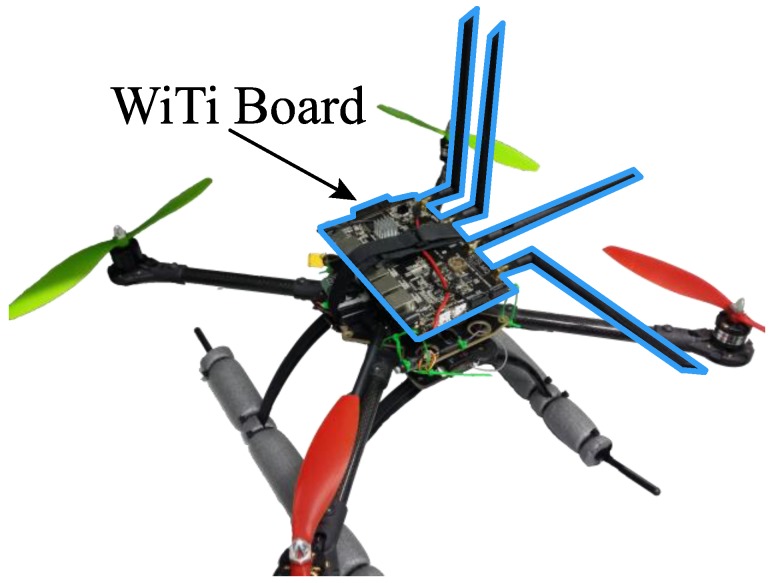
UAV and WiTi device.

This section details the hardware, software, and test bench used to study the complete radiation pattern in a fully-controlled experimental environment. The UAV used in the experiments was the IdeaflyIFLY-4S (Shenzhen Idea-Fly Technology Co.,Ltd, ShenZhen, China), a quadcopter with carbon fiber chassis, brushless motors, and interchangeable plastic propellers. A wireless communication module was added to this UAV; specifically, the open firmware router WiTi Board (hereinafter WiTi, mqmaker, Shenzhen, China) [66]. It is based on OpenWRT v14.07 with dual band and two antenna connectors for each band, thus allowing the connection of up to four antennas at the same time. The communication chipsets were the MT7602E that supports 802.11 b/g/n WiFi 2T2R and the MT7612E that supports 802.11 a/b/g/n/ac WiFi 2T2R, using the 2.4 GHz and 5 GHz bands, respectively. Both chipsets can work independently and concurrently. Figure 9 shows the UAV and the WiTi device. Two types of dual-band antennas were employed, namely, antennas with an omnidirectional radiation pattern and 5 dBi gain in both bands (ARS-NT5B, Alfa Network, Taipei, Taiwan) and directional patch antennas with a gain of 8 dBi for 2.4 GHz and 10 dBi for 5 GHz (APA-M25, Alfa Network, Taipei, Taiwan). The R&S ZVL spectrum analyzer (Rohde & Schwarz, Inc., Columbia, MD, USA) was used in reception to capture signals up to 6 GHz, along with the R&S receiver antenna HF906 (Rohde & Schwarz, Inc., Columbia, MD, USA) with a large reception bandwidth (between 1 GHz and 18 GHz) and a constant gain (10 dBi for 2.4 GHz and 11 dBi for 5 GHz). Finally, the employed RF anechoic chamber was a full chamber with isolation cones that prevent electromagnetic waves from bouncing off walls, ceilings, and floors. The dimensions of the RF anechoic chamber were 5.2 m × 3.5 m × 2.25 m. The rotary table INN-CO DE 3260-P was located inside the chamber and controlled by the digital controller INN-CO CO 2000.

**Figure 10:**
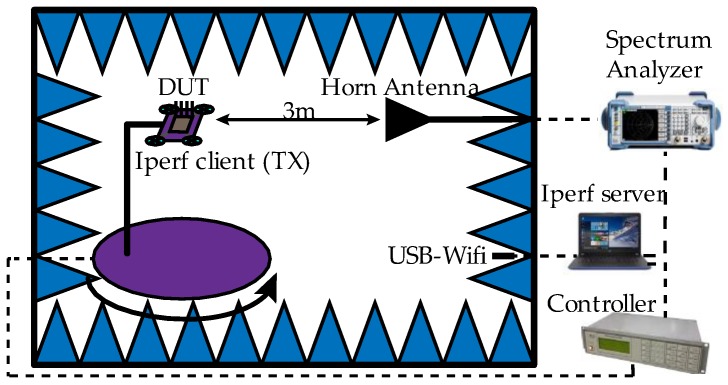
Experimental testbed elements (DUT ≡ Device Under Test ≡ UAV + WiTi device).

The UAV with the embedded WiTi were placed on the rotary table. The WiTi was configured to act as a transmitter (client) in a session of data transmission. To this end, the traffic generator iperf v2.0.9 [10] was used in its default configuration to send TCP (transport control protocol) traffic. A USB-WiFi device located inside the RF anechoic chamber and controlled by a computer from outside the chamber was used as the server of the session. While the rotary table was rotating, the ZVL spectrum analyzer connected to the HF906 antenna captured the power transmitted by the WiTi. Power measurements were taken every 10 degrees, in a bandwidth of 20 MHz centered on the center frequency with a resolution bandwidth of 3 kHz, thus eliminating the limits of the working band and remaining in a flat spectrum in frequency. The captured power was averaged and a value representing the power received for each rotation angle (36 in total) was obtained at a distance of 3 m and a height of 1.5 m (fulfilling the condition of far field for both working bands and both antennas). The complete test bench scenario is shown in Figure 10. Table 5 summarizes the test bench parameters.

### 3.3. Results

We conducted three sets of experiments whose description and results are explained as follows. The first set of experiments aimed to get the best configuration of the antennas for the WiTi as an isolated device, i.e., the most appropriate orientation in order to achieve the highest signal power. This was done for the 2.4 GHz band and the 5 GHz band, which corresponded to the U2U and U2G links, respectively, as shown in Figure 1.

**Figure 11:**
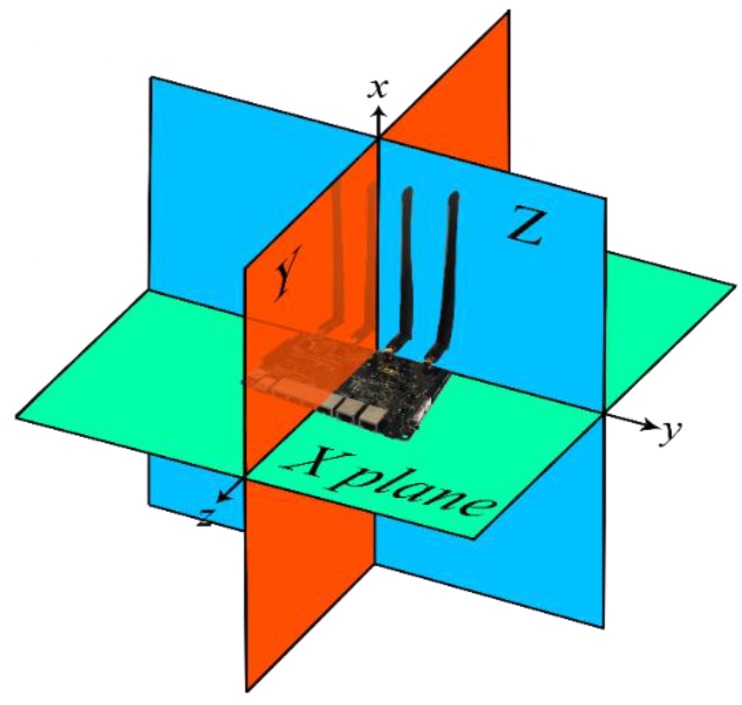
Planes under study.

**Figure 12:**
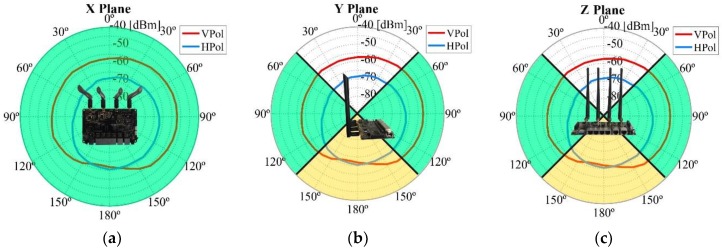
Example of a measurement of the complete radiation pattern and example of possible areas of interest for each communication link, which are highlighted in green for the U2U link and yellow for the U2G link: (**a**) X Plane, (**b**) Y Plane, and (**c**) Z Plane.

To obtain the radiation pattern as accurately as possible, three planes were studied, depicted in Figure 11 as X Plane, Y Plane, and Z Plane. Within each plane, both vertical and horizontal polarizations were studied. Figure 12 depicts a detailed example of a measurement and represents how the WiTi appeared on each spatial cut (X, Y, and Z planes). The regions of interest for the U2U and U2G links are also highlighted in green and yellow colors in Figure 12, respectively. These regions of interest were used to select the best configuration of the antennas considering a specific scenario. Particularly, we assumed that for communications among UAVs (U2U links), the regions of interest should be located in the same horizontal plane (±45°). Therefore, the region of interest for communication among UAVs corresponds to the complete X plane plus two areas of 90° (from 45° to 135° and from −45° to −135°) seen from the Y and Z planes as shown in Figure 12. On the other hand, we set the region of interest for U2G communications as a 90° coverage (from 135° to −135°) in Y and Z planes from the UAV to the ground. Therefore, the optimal antenna configuration was such that the maximum power was obtained in the so-defined regions of interest. Please observe that for another type of network deployment, e.g., in case of a satellite-aided UAV communication, the region of interest for the UAV-satellite link would be different, being maybe interested in that case in using the antennas configuration that would maximize power transmission in Y and Z planes with an angle of 90° (from −45° to 45°).

**Figure 13:**
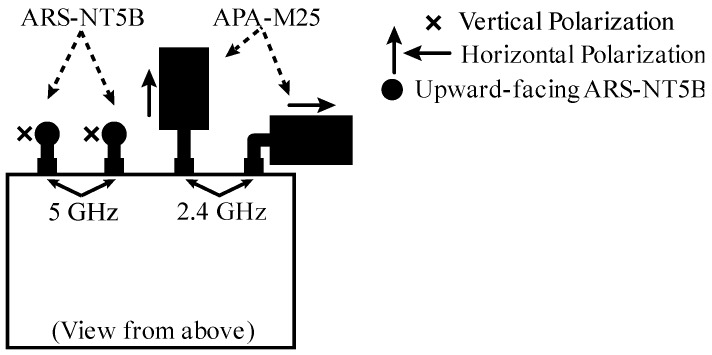
The best antenna configurations of the WiTi as seen from above: 2.4 GHz band for the U2G link with APA-M25 antennas in horizontal position with 90° between them and 5 GHz band for the U2U link with ARS-NT5B antennas in vertical position.

**Figure 14:**
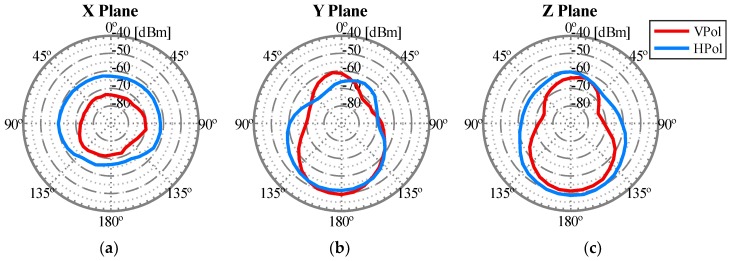
Radiation pattern of the isolated WiTi using the antennas configuration shown in Figure 13 in the U2G link working in 2.4 GHz: (**a**) X Plane, (**b**) Y Plane, and (**c**) Z Plane.

**Figure 15:**
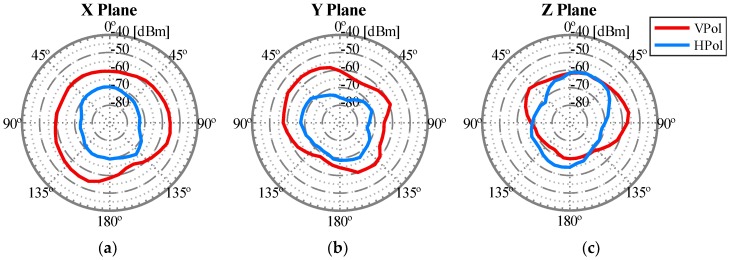
Radiation pattern of the isolated WiTi using the antennas configuration shown in Figure 13 in the U2U link working in 5 GHz: (**a**) X Plane, (**b**) Y Plane, and (**c**) Z Plane.

After carrying out this first set of experiments, the best antenna configuration that we observed is shown in Figure 13. The corresponding radiation pattern is depicted in Figure 14 for the 2.4 GHz band and in Figure 15 for the 5 GHz band. For example, note that with the configuration shown in Figure 13 we obtained the maximum power transmitted in the region of interest defined for the Y plane (from 135° to −135°). These results could be considered as expected given that to optimize the U2G link, the power must be transmitted from the UAV downwards, and this is achieved by orienting the antennas towards the nadir. In addition, if the antennas rotate 90 degrees between them, we have polarization diversity and, therefore, we avoid a situation of crossed polarization between the receiving antenna and any of the transmitting antennas at the same time. Another important feature was the gain of the antennas. For this U2G link, it must be adjusted to achieve a main lobe width of about 90 degrees, which allowed us to adjust it to the region of interest from 135° to −135°. This gain presented its optimal value for the 2.4 GHz band with a gain of 8 dBi, compared to 10 dBi in the 5 GHz band. However, the 5 GHz option would provide a narrower lobe that did not fit our region of interest. On the other hand, for the U2U links, since an omnidirectional radiation pattern was necessary in the X plane (see Figure 12), the best results were obtained with the ARS-NT5B antennas in a vertical position, as can be seen in Figure 13.

The goal of the second set of experiments was to study the influence (if any) of the UAV chassis, the UAV motors (RPMs and vibrations produced in antennas and chassis), and the UAV propellers on the radiation pattern in both the 2.4 GHz and 5 GHz bands. To do so, the WiTi was located on-board the UAV, and continued operating as a client, thus transmitting data to the server device. Once inside the RF anechoic chamber, we took measurements in the following states: UAV off, UAV on with engines at 45% of its power with and without propellers, UAV on at 65% power without propellers, and UAV on at 95% power without propellers. The results obtained indicate that the influence of the drone in the radiation pattern was mainly due to the chassis. The impact of propellers, of the power of the engines, and of the vibration produced by the engines was negligible in our study. In our opinion, all the system is equilibrated without vibrations because we were using a brushless motor and the propellers were made of plastic. This result could be different if carbon fiber propellers were used, which requires further work.

**Figure 16:**
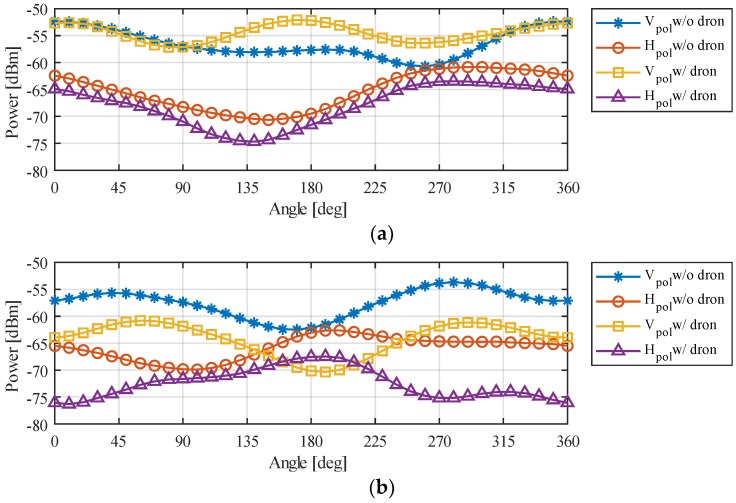

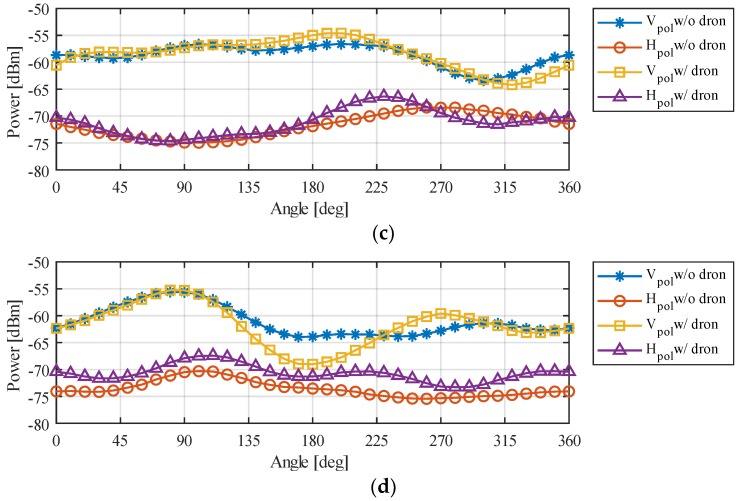
Influence of the UAV on the radiation pattern in: (**a**) 2.4 GHz band and X Plane, (**b**) 2.4 GHz band and Y Plane, (**c**) 5 GHz band and X Plane, and (**d**) 5 GHz and Y Plane.

Regarding the effect of the UAV chassis, it was more notable in the Y and Z planes. Thus, the U2G link (Planes Y and Z) was more affected, with a variation of the received power of up to −10 dB in the 2.4 GHz band (the band utilized for the U2G link), which means that the power falls to a tenth of the original value in both working bands. Regarding the X plane in the 2.4 GHz band, we could observe a −5 dB in some angles, reducing the power to almost a quarter of its original value. Figure 16 illustrates the change in the radiation pattern when the WiTi was used alone or when it was used as a communication device on-board the UAV in the 2.4 GHz band in the X plane (see Figure 16a) and in the Y plane (see Figure 16b), and in the 5 GHz band in the X plane (see Figure 16c) and in the Y plane (see Figure 16d). The results obtained in the Z plane were identical to the Y plane. From these observations, incorporating the influence of UAV in propagation models could be done by modeling the UAV as additional system losses. For UAVs like the one employed in this study, the system loss was equivalent to −10 dB on the U2G link and −5 dB for the U2U link. By doing so, we could guarantee that the power received in each of the bands would always be equal to or greater than the calculated one if the limits of the regions of interest delimited in Figure 12 are respected.

**Figure 17:**
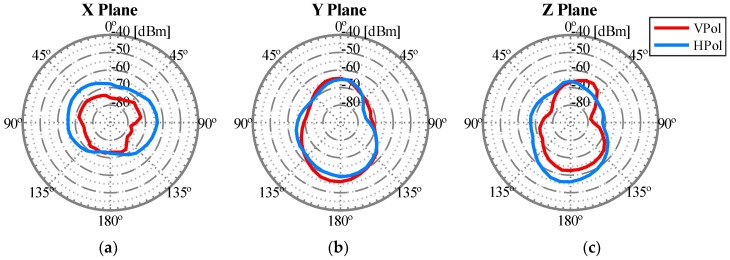
Radiation pattern of the WiTi on-board the UAV for the antennas configuration shown in Figure 13 in the U2G link working in 2.4 GHz: (**a**) X Plane, (**b**) Y Plane, and (**c**) Z Plane.

**Figure 18:**
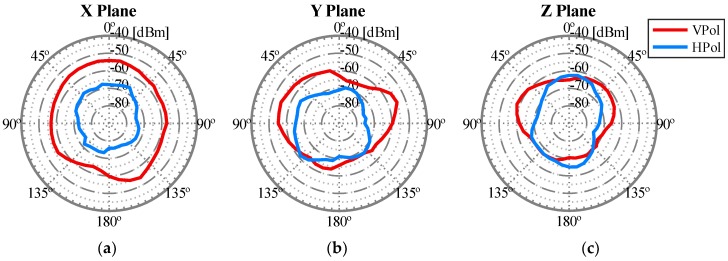
Radiation pattern of the WiTi on-board the UAV for the antennas configuration shown in Figure 13 in the U2U link working in 5 GHz: (**a**) X Plane, (**b**) Y Plane, and (**c**) Z Plane.

Once it was demonstrated that the influence of the UAV was only due to the chassis, the aim of the last set of experiments was to obtain the most accurate radiation pattern of the whole system using the optimal antennas configuration for our assumed network deployment, i.e., the configuration of the antennas that offered the highest radiated power for each communication link U2U and U2G. The outcomes obtained are shown in Figure 17 and Figure 18, representing the real radiation patterns that should be considered for an effective network deployment. Furthermore, with this configuration of antennas, it can be assumed that the radiation diagram produced by the UAV was isotropic for the U2U link and non-isotropic but constant for the U2G link, both within the regions of interest described above for each communication link.

## 4. Conclusions

Along this work, we reviewed the state of the art related to mobility, positioning, and propagation models in flying ad hoc networks. While we detected a wide variety of contributions in mobility, positioning and propagation models still require further work from the research community to face the imposed challenges. One of these necessary improvements comprehends the use of FANETs to extend telecommunications coverage. The network communication capability of UAVs is usually provided by on-board communication devices that include one or several wireless technologies (e.g., WiFi, WiMAX, LTE, etc.). Even though these technologies are well known, quantifying the influence of the UAV on the radiation pattern of the on-board communication devices is still under study. Therefore, we also presented a case study in which we measured this impact in a fully-controlled environment, an RF anechoic chamber. First, we obtained the best position of the antennas for a WiFi communication device taking into account that it will be an aerial node. Whereas communications are usually focused in a 2-D plane, this new type of aerial communication must offer a reliable 3-D reliable coverage. Specifically, we obtained the best configuration of antennas for a U2U link and for a U2G link working at the 2.4 GHz and 5 GHz bands, respectively. Afterwards, we evaluated the influence of the UAV device on the radiation patterns, concluding that the influence of the UAV chassis represented a decrease in the transmitted power of around 10 dB for the U2G link, which meant that the power fell to a tenth of the original value. Finally, we demonstrated that the radiation pattern of a communication device on-board a UAV should not be approximated by an isotropic diagram unless (i) the correct orientation of the antennas is used and (ii) ensuring that, if the radiation diagram is not isotropic, at least the regions of interest have a constant radiation as we showed in this study.

## Figures and Tables

**Table 1:** Classification of UAVs by size.

Type of UAV		Speed	Energy Autonomy	Mobility Degree	Static Hover	Altitude
Large-UAV	FW	High	High	Low	No	High
	RW	Low-Med	Low-Med	Med-High	Yes	Med-High
Small-UAV	FW	Med-High	Med-High	Low-Med	No	Low-Med
	RW	Low	Low	High	Yes	Low

**Table 2:** Comparison of MANET, VANET, and FANET.

Characteristics	MANET	VANET	FANET
Node Mobility	Lower (2-D)	Low (2-D)	RW-UAV: High (3-D) FW-UAV: Medium (3-D)
Node Speed	Lower (6 km/h)	Medium–High (20–100 km/h)	RW-UAV: Medium (50 km/h) FW-UAV: High (100 km/h)
Mobility Model	Random	Manhattan models	RW-UAV: RWP [17] FW-UAV: PPRZM [18]/ST [19]
Topology Change	Low	Medium	High
Energy Constraints	Medium	Low	RW-UAV: High (15–30 min) FW-UAV: Medium (to 5 h)

**Table 3:** The taxonomy of mobility models, the adequate type of UAV, and examples of their applications.

Class	Ref	Mobility Model	RW/FW UAV	Applications
Pure Randomized	[21]	RW	RW	Environmental sensing/Traffic and urban Monitoring
	[17]	RWP	RW	
	[22]	RD	RW	
	[23]	MG	RW	
Time-Dependent	[24]	BSA	RW	Environmental sensing/Search and rescue
	[25]	GM	RW	
	[26]	EGM	RW/FW	
	[27]	3D-GM	RW/FW	
	[19]	ST	RW/FW	
Path-Planned	[28]	SRCM	RW	Agricultural management/Traffic and urban Monitoring
	[18]	PPRZM	RW/FW	
Group	[35]	ECR	RW	Environmental sensing/Search and rescue
	[31]	PSMM	RW/FW	
	[32]	RPGM	RW	
	[21]	CLMN	RW	
	[21]	NC	RW	
	[21]	PRS	RW/FW	
Hybrid	[34]	H3MP	RW	Surveillance/Search and rescue

**Table 4:** Comparative of channel models in literature.

Ref.	Type	Channel	Frequency (GHz)
[16]	Theoretical	UAV–Ground	2.4
[54]	Theoretical	UAV–Ground	2–6
[55]	Empirical	UAV–Ground	1.8–5.76
[56]	Empirical	UAV–Ground	0.8
[57]	Empirical	UAV–Ground	0.96–0.977
			5.030–5.091
[58]	Empirical	UAV–Ground	3.1–5.3
[59]	Semi-Empirical	UAV–Ground	2
[13]	Semi-Empirical	UAV–UAV	2.4

**Table 5:** Summary of parameters and characteristics of the test bed.

Device or Parameter	Configuration
TX Antennas (Antenna Gain 2.4/5 GHz)	ARS-NT5B (5/5 dBi), APA-M25 (8/10 dBi)
RX Antenna (Antenna Gain 2.4/5 GHz)	HF906 (10/11 dBi)
Distance between TX and RX Antennas	3 m
Traffic Generator	Iperf v2.0.9 (TCP default configuration)
Center Frequency	2.462/5.210 GHz
Transmitted BW	40/40 MHz
Captured BW	20/20 MHz
TX Power	20/20 dBm
